# Supplementary material for: A stratospheric precursor of East Asian summer droughts and floods
Source: Nat Commun. 2024 Jan 4;15:247. doi: 10.1038/s41467-023-44445-y (PMC10764360; doi:10.1038/s41467-023-44445-y)
Supplement: Supplementary file 1 — Supplementary Information [file 41467_2023_44445_MOESM1_ESM.pdf]

# Supplementary Information

# A stratospheric precursor of East Asian summer droughts and floods

Ruhua Zhang[a], Wen Zhou[a*], Wenshou Tian[b], Yue Zhang[a],

Junxia Zhang[c], Jiali Luo[b]

[a]*Key Laboratory of Polar Atmosphere-ocean-ice System for Weather and Climate, Ministry of Education, Department of Atmospheric and Oceanic Sciences and Institute of Atmospheric Sciences, Fudan University, Shanghai, China*

[b]*Key Laboratory for Semi-Arid Climate Change of the Ministry of Education, College of Atmospheric Sciences, Lanzhou University, Lanzhou, China*

[c]*Lanzhou Central Meteorological Observatory of Gansu Province, Lanzhou, China*

*Corresponding author: Wen Zhou, wen_zhou@fudan.edu.cn*

**Abstract** This document provides supplementary information to "A stratospheric precursor of East Asian summer droughts and floods", including three Tables and 10 Figures, which has been mentioned in the main manuscript.

## Experimental design

We use Community Atmosphere Model version 6 (CAM6) in the Community Earth System Model Version 2.1.3 (CESM2) to design nudging experiments. Although the CAM6 model has a low top and poor performance in stratospheric simulations, it does not influence the reliability of our results, due to the whole stratospheric nudging technology. The stratospheric conditions (*u*, *v*, T) above the 143 hPa model level are nudged to the JRA-55 reanalysis datasets with a maximal nudging coefficient (1.0). Thus, stratospheric conditions are very close to the observations. We can then see the responses of precipitation to the historical QBO.

**Supplementary Table 1** Stratospheric nudging experiment design

| | |
|---|---|
| E0 | Control experiment is forced by the climatological fields (1995-2005, the default setting in the F2000climo component) and runs for 20 years, using CAM6 of CESM2 with 32 vertical levels and a horizontal resolution of 1.9°×2.5° (latitude×longitude). The first 5 years are spin-up time, and the remaining 15 years are used to force the sensitivity experiments as initial conditions (atmospheric states on 1st March). |
| E1 | As in E0, but the whole stratospheric conditions (u, v, T) above 143 hPa are nudged to the JRA-55 reanalysis datasets with a nudging coefficient of 1.0 (4 times per day). The levels between 143 and 198 hPa are set as the transition layer with nudging coefficients decaying from 1.0 to 0. No nudging is used below the 198 hPa model level. This whole stratospheric nudging avoids the poor simulation of stratospheric conditions in the models. Fifteen sets of nudging experiments are run using different initial conditions (atmospheric states on 1$^{st}$ March in 6-20 years) from E0. Each set of nudging experiments runs for 6 months (March to August) every year from 1995 to 2019. |
| E1 parameters | nudge_hwin_lat0 = 0 & nudge_hwin_lon0 = 180.<br>nudge_hwin_latdelta = 0.001 & nudge_hwin_londelta = 0.001<br>nudge_hwin_latwidth = 999 & nudge_hwin_lonwidth= 999<br>nudge_vwin_hdelta = 0.2 & nudge_vwin_hindex = 14. |

**Supplementary Table 2** Correlation coefficients among different factors

| Cor | $QBO_{10\text{-}hPa}$ | $QBO_{70\text{-}hPa}$ | NTA | SNA | SIO | Niño 3.4 | DMI |
|---|---|---|---|---|---|---|---|
| Niño 3.4 | 0.37 | -0.18 | -0.47 | -0.22 | 0.10 | 1 | **0.47** |
| DMI | **0.64** | **-0.46** | -0.48 | -0.47 | 0.43 | **0.47** | 1 |

**Supplementary Table 3** Multicollinearity of different factors

| | $QBO_{10\text{-}hPa}$ | NTA | SNA | SIO | $QBO_{70\text{-}hPa}res$ |
|---|---|---|---|---|---|
| Tolerance | 0.903 | 0.716 | 0.897 | 0.912 | 0.769 |
| Variance inflation factor | 1.107 | 1.397 | 1.115 | 1.096 | 1.300 |

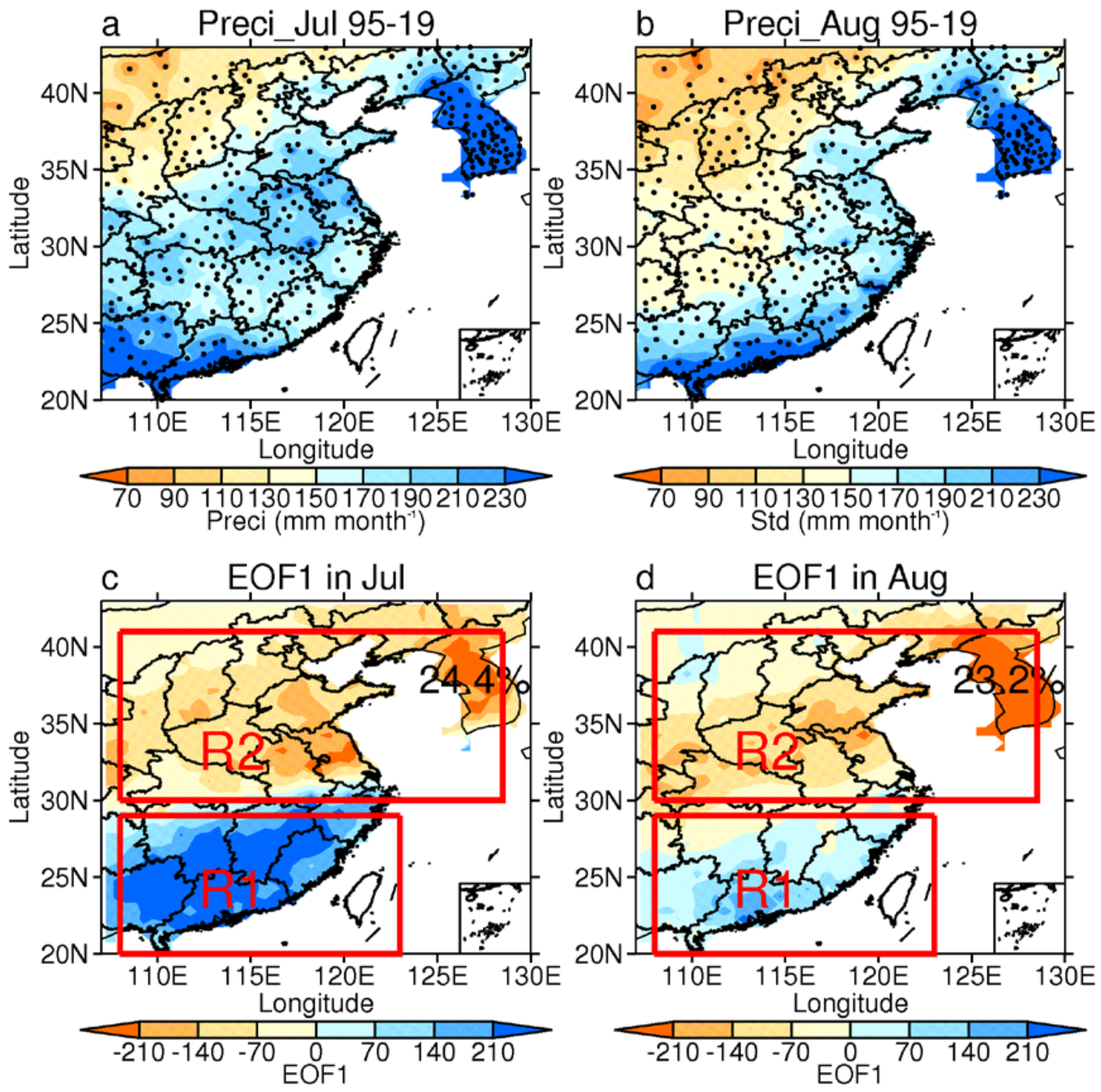


**Supplementary Fig.1. East Asian precipitation (107-130°E, 20-43°N) in July and August over the period 1995–2019. a** Spatial distribution of climatological precipitation in July. **b** Spatial distribution of climatological precipitation in August. **c** EOF1 in July. **d** EOF1 in August. Black dots in **a-b** indicate the location of stations. The precipitation data are interpolated onto 0.5×0.5 (longitude×latitude) grids.

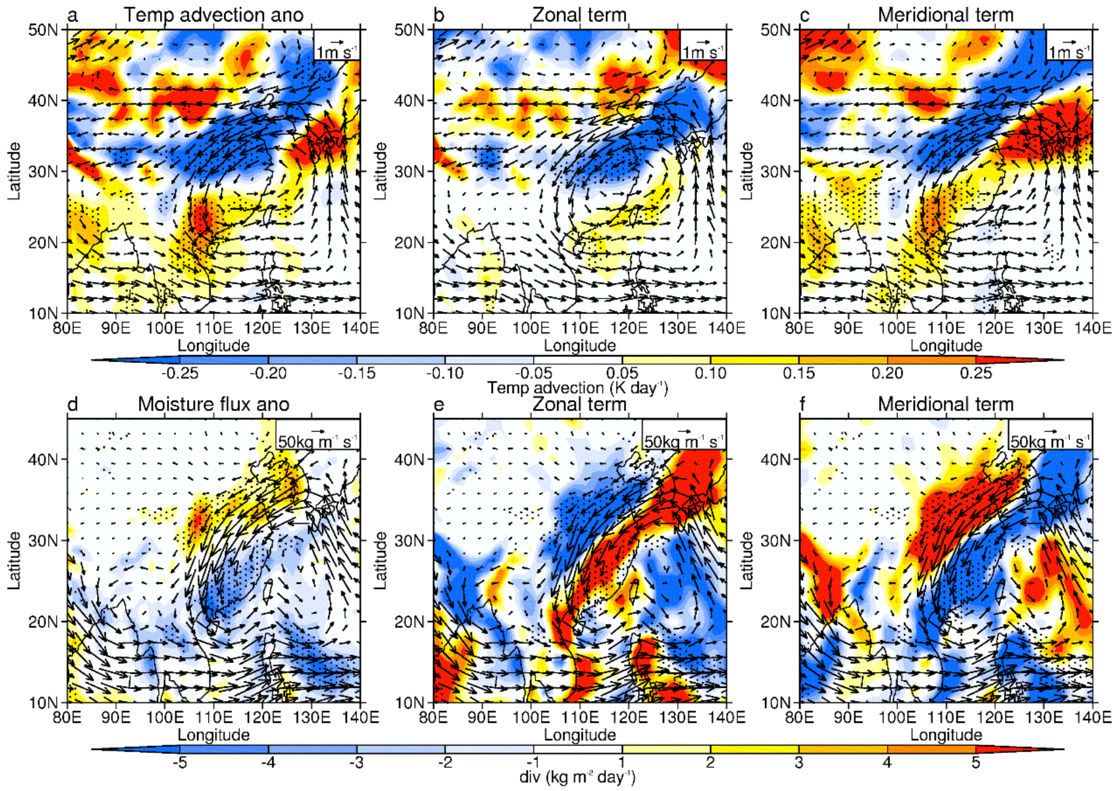


**Supplementary Fig.2. Composite differences (SFND minus SDNF) of circulation anomalies.** **a** Horizonal wind (vectors) and temperature advection (colors) anomalies at 500 hPa. **b** Horizonal wind (vectors) and zonal term of temperature advection (colors) and anomalies at 500 hPa. **c** Meridional term of temperature advection (colors) and horizonal wind (vectors) anomalies at 500 hPa. **d** Vertically integrated moisture flux (vectors) and its divergence (colors) anomalies. **e** Vertically integrated moisture flux (vectors) and zonal term of its divergence (colors) anomalies. **f** Vertically integrated moisture flux (vectors) and meridional term of its divergence (colors) anomalies. Dots indicate values at the 95% confidence level.

The anomalous warm (cold) temperature advection in the south (north) side of 30°N (Supplementary Fig.2a), corresponding to the vertical motion in Fig. 2b. Both the zonal term and meridional term make positive contributions to the temperature advection anomalies (Supplementary Fig.2b-c). For the moisture flux, meridional water vapor transport plays the most important role (Supplementary Fig.2d-f).

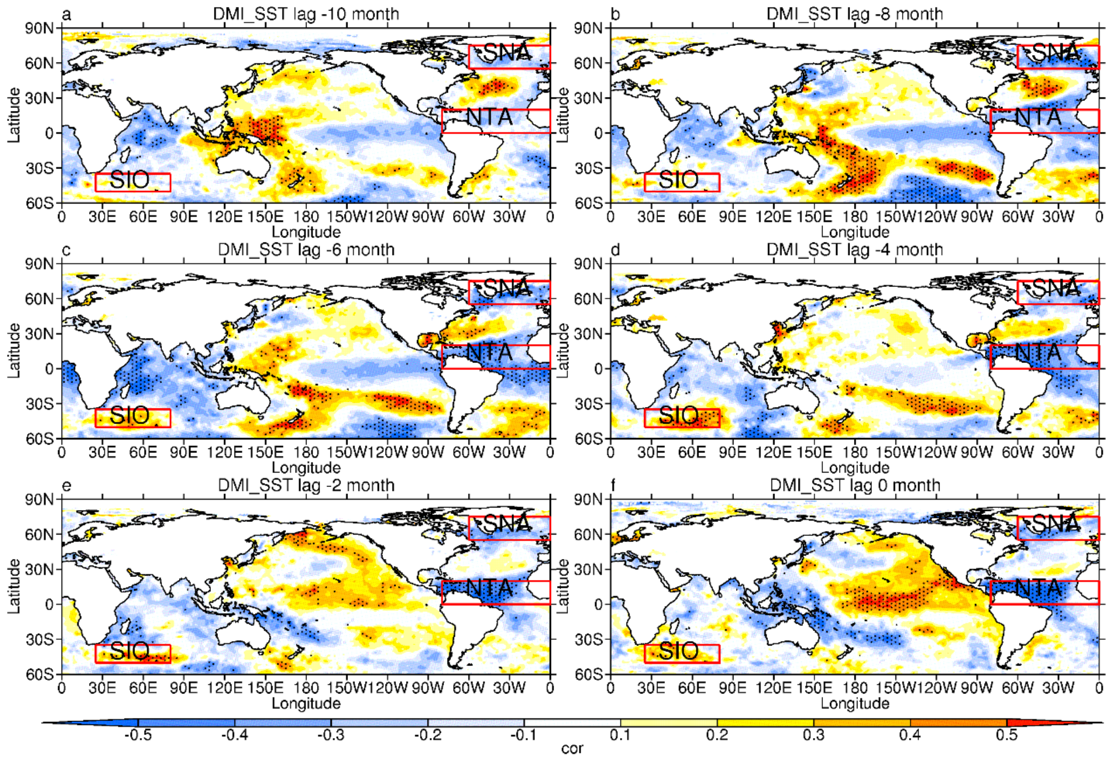


**Supplementary Fig.3. Lag-correlation coefficients between the DMI in July-August and detrended SST anomalies. a** 10-month lag-correlation coefficients **b** 8-month lag-correlation coefficients **c** 6-month lag-correlation coefficients. **d** 4-month lag-correlation coefficients. **e** 2-month lag-correlation coefficients. **f** Simultaneous correlation coefficients. The SST anomalies precede the DMI in **a-e**, and dots indicate values at the 95% confidence level.

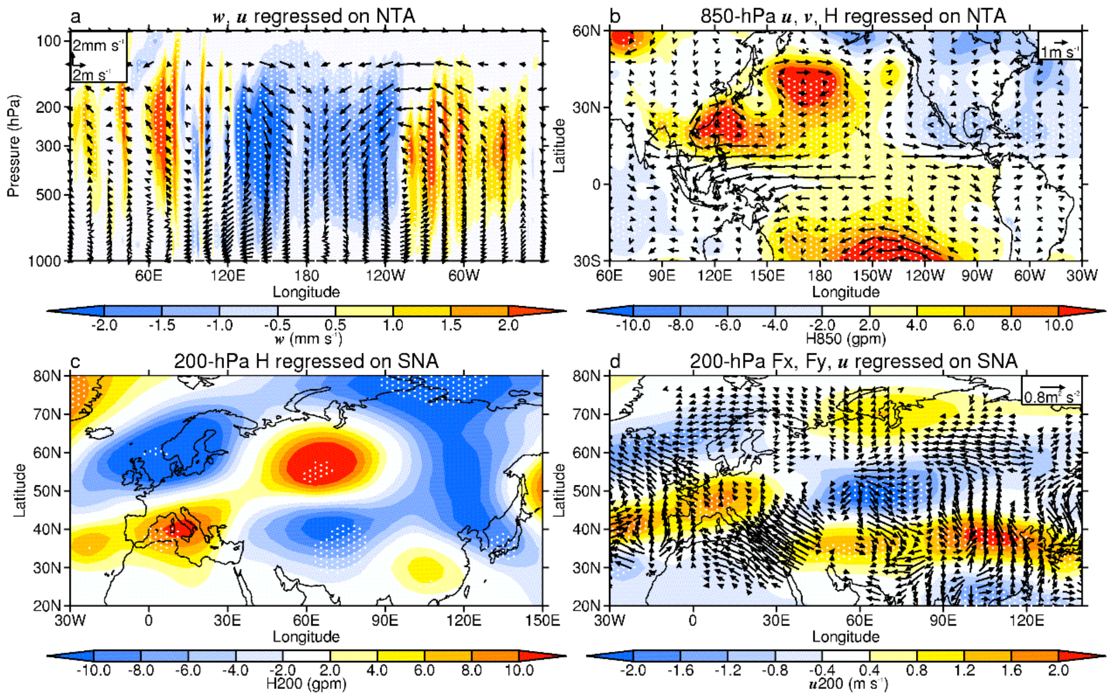


**Supplementary Fig.4. Circulation anomalies regressed on the standardized NTA and SNA SST. a** Subtropical vertical velocity (colors) and zonal circulation (vectors) anomalies averaged over 5°N-20°N regressed on the NTA index. **b** The 850-hPa wind (vectors) and geopotential height (colors) anomalies regressed on the NTA index. **c** The 200-hPa geopotential height (colors) anomalies regressed on the SNA index. **d** The 200-hPa zonal wind (colors) and wave flux (vectors) anomalies. Dots indicate values at the 95% confidence level.

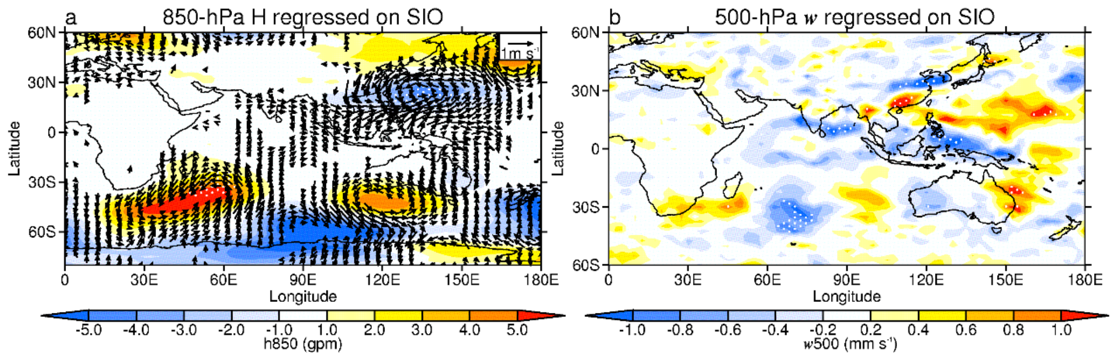


**Supplementary Fig.5. Circulation anomalies regressed on the standardized SIO index. a** The 850-hPa wind (vectors) and geopotential height (colors) anomalies. **b** The 500-hPa vertical velocity anomalies. The linear effects of ENSO (Niño 3.4) and the southern annular mode have been removed in the anomalies. Dots indicate values at the 90% confidence level. The Niño-3.4 index is computed as the area-averaged SST anomalies (5°S-5°N, 170°-120°W). The southern annular mode index is defined as the difference in surface pressure between 40°S and 65°S.

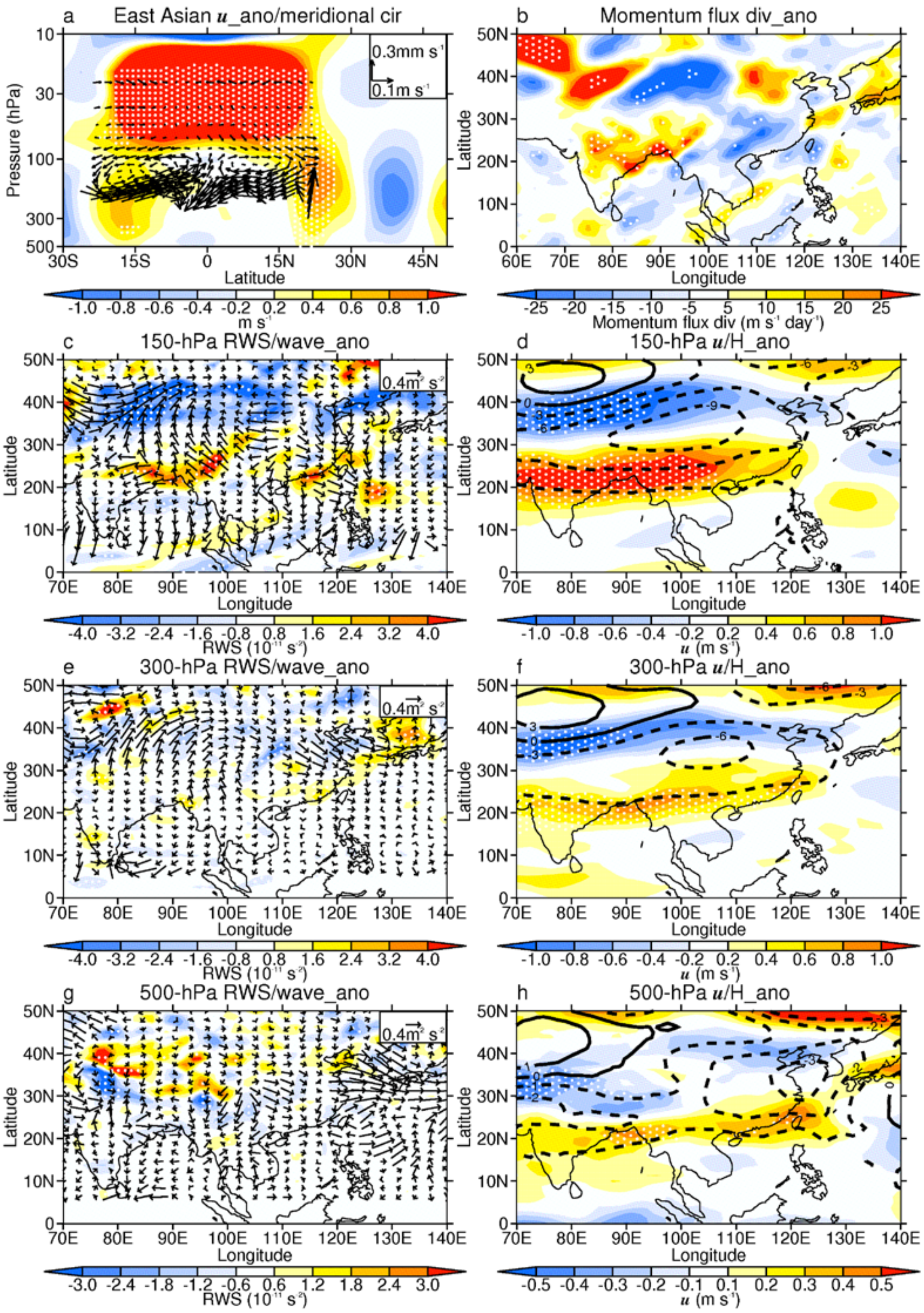


**Supplementary Fig.6. East Asian circulation anomalies regressed on the standardized 30-hPa QBO index. a** Zonal wind (colors) and meridional circulation (vectors) anomalies averaged over 70°-120°E. **b** The momentum flux (-*u'v'*) divergence anomalies at 200 hPa. **c** The 150-hPa Rossby wave source (colors) and Plumb wave flux (vectors) anomalies. **d** The 150-hPa zonal wind (colors) and geopotential height (contours) anomalies. **e** and **g** As in **c** but for 300 hPa and 500 hPa, respectively. **f** and **h** As in **d** but for 300 hPa and 500 hPa, respectively. The units of geopotential height are gpm in **d, f, h**. The *u'* and *v'* are the deviations from the zonal mean zonal wind and meridional wind, respectively. Linear effects of NTA, SNA, and SIO have been removed, and dots indicate values at the 90% confidence level.

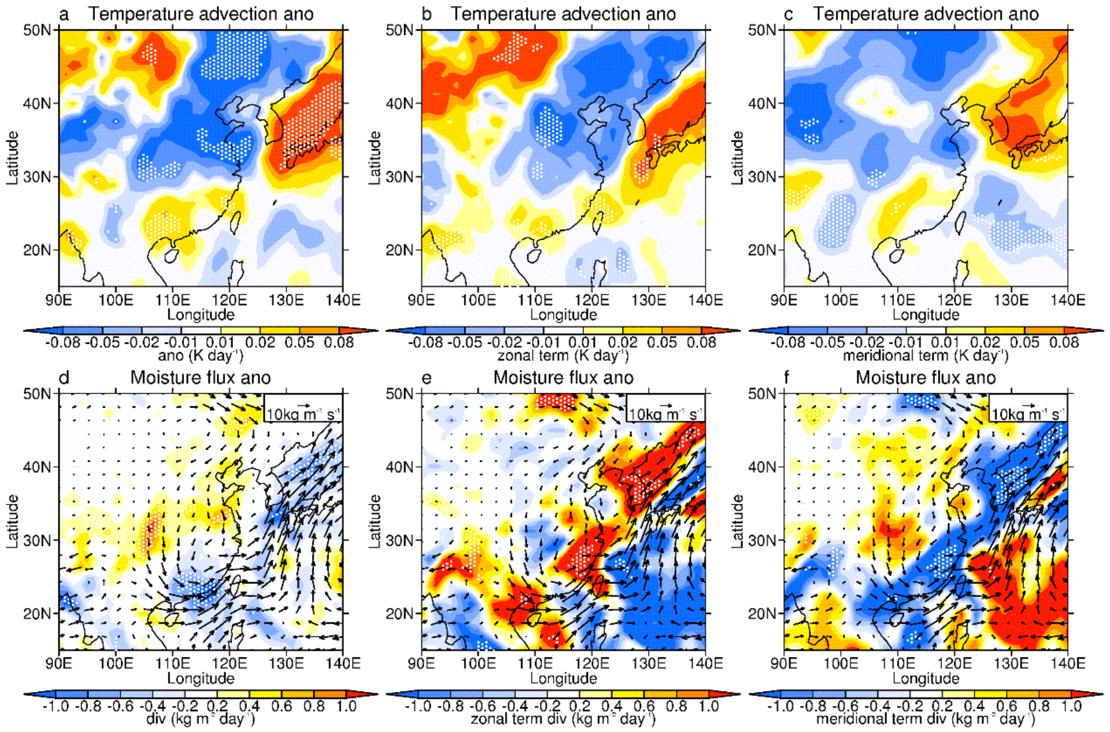


**Supplementary Fig.7. Temperature advection and moisture flux anomalies regressed on the standardized 30-hPa QBO index. a** Temperature advection anomalies (colors) at 500 hPa. **b** Zonal term of temperature advection anomalies (colors) at 500 hPa. **c** Meridional term of temperature advection anomalies (colors) at 500 hPa. **d** Vertically integrated moisture flux (vectors) and its divergence (colors) anomalies. **e** Vertically integrated moisture flux (vectors) and zonal term of its divergence (colors) anomalies. **f** Vertically integrated moisture flux (vectors) and meridional term of its divergence (colors) anomalies. The linear effects of NTA, SNA, and SIO have been removed, and dots indicate values at the 90% confidence level.

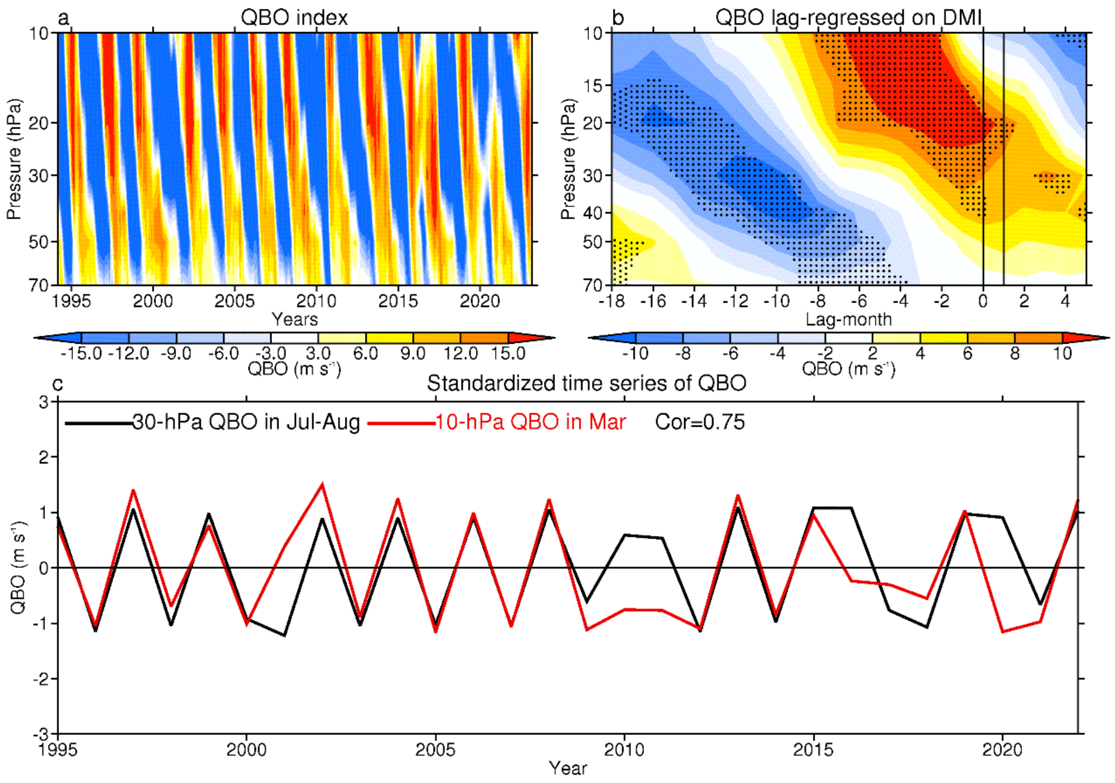


**Supplementary Fig.8. Downward extension of the QBO signal. a** Temporal development of the QBO index. **b** The QBO anomalies lag-regressed on the DMI index. **c** Time series of the 30-hPa QBO index in July-August and the 10-hPa QBO index in March. Straight lines in (b) are July-August, and dots indicate values at the 95% confidence level.

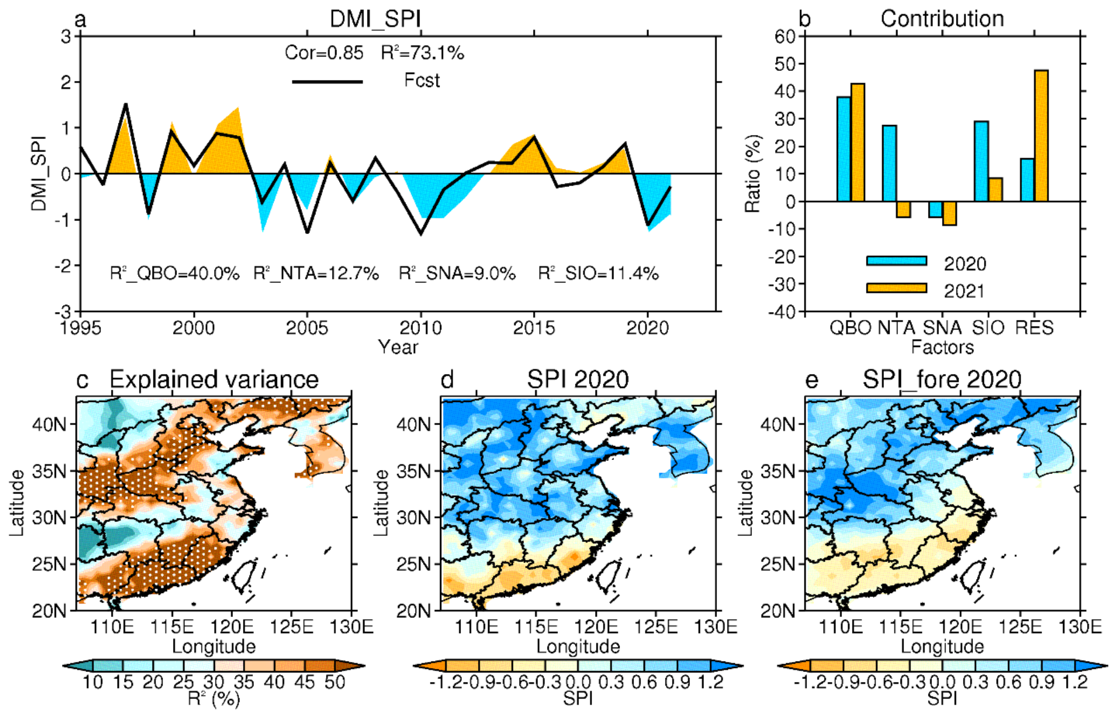


**Supplementary Fig.9. As in Fig. 5b-f but for SPI.**

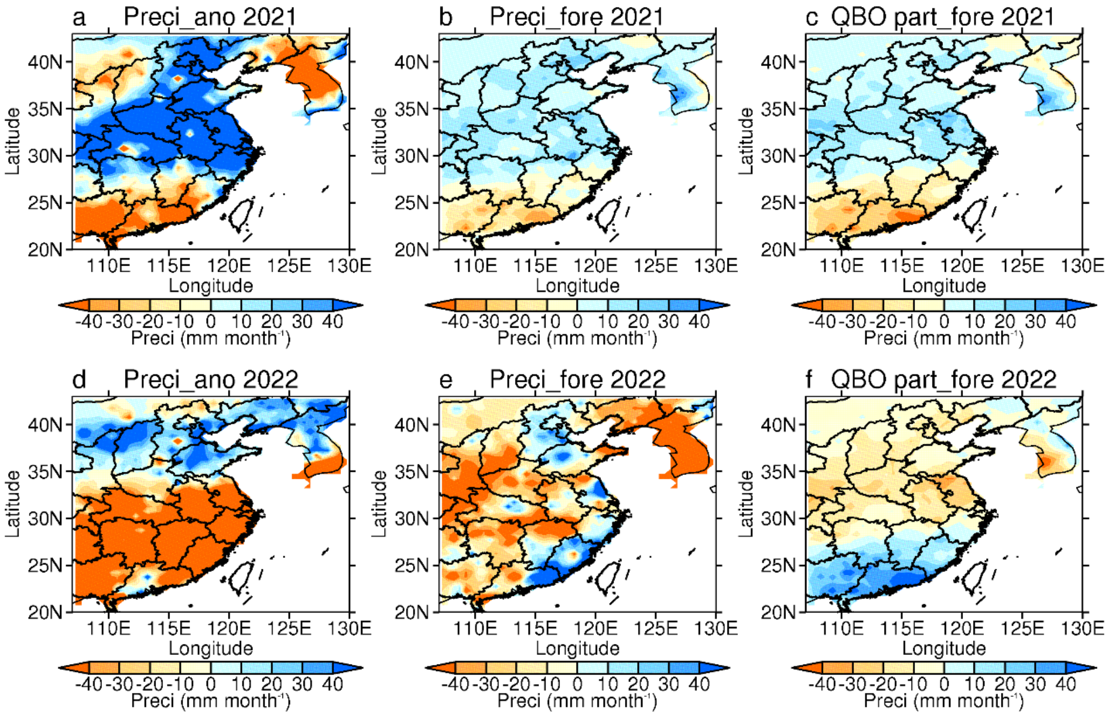


**Supplementary Fig.10. Seasonal forecasting for July-August precipitation over East Asia. a** Observational precipitation anomalies in 2021. **b** As in **a** but for forecasting results in 2021. **c** The QBO-induced parts of forecasting results in 2021. **d-f** As in **a-c** but in 2022.
